# Nexilin mutations are associated with left ventricular noncompaction cardiomyopathy

**DOI:** 10.1186/2194-7791-2-S1-A7

**Published:** 2015-07-01

**Authors:** E Pardun, K Wenzel, H-H Kramer, F Berger, B Gerull, S Klaassen

**Affiliations:** Dept. of Pediatric Cardiology, ECRC, University Medicine-Charité, Berlin, Germany; Dept. of Congenital Heart Disease, University Clinic, Kiel, Germany; University of Calgary, Calgary, Alberta Canada

## Introduction

Left Ventricular Noncompaction Cardiomyopathy (LVNC) is a very rare congenital heart disease. LVNC is a form of cardiomyopathy in which the fetal myocardium fails to "compact" during cardiac development and it may be associated with impairment of LV function and LV dilatation. Mutations in several sarcomere genes have been described in LVNC. Nexilin, encoded by *NEXN*, is a cardiac Z-disc protein that stabilizes the sarcomere. We evaluated nexilin as a disease gene for LVNC.

## Methods

Ninety-two LVNC patients and 254 controls were screened and *NEXN* was sequenced by Sanger sequencing.

## Results

We found a missense mutation (c.1408 G>C, p.Glu470Gln) in one patient and a nonsense mutation in another patient (c.1723G>T, p.Glu575*). None of these mutations were detected in the healthy controls. In addition, we identified one single nucleotide polymorphismus (c.733G>A, p.Gly245Arg) homozygous in two and heterozygous in three LVNC patients. This polymorphism was also found in 78 controls (homozygous in 12 and heterozygous in 64 controls).

## Conclusion

Mutations in genes encoding Z-disc proteins such as nexilin have been shown to cause different forms of cardiomyopathy. Therefore, the two mutations we identified in *NEXN* may further increase our knowledge of Z-disc genes in the pathogenesis of LVNC. To establish the disease causality, it is necessary to investigate the effect of the mutations on protein function in further in vitro studies.

